# Supervised Machine Learning to Examine Factors Associated with Respiratory Sinus Arrhythmias and Ectopic Heart Beats in Adults: A Pilot Study

**DOI:** 10.3390/hearts5030020

**Published:** 2024-07-05

**Authors:** Peyton Lahr, Chloe Carling, Joseph Nauer, Ryan McGrath, James W. Grier

**Affiliations:** 1 Department of Health, Nutrition, and Exercise Sciences, North Dakota State University, Fargo, ND 58108, USA; 2 College of Osteopathic Medicine, Rocky Vista University, Parker, CO 80112, USA; 3 Department of Biological Sciences, North Dakota State University, Fargo, ND 58108, USA

**Keywords:** electrocardiography, heart, lifespan, risk

## Abstract

**Background:**

There are many types of arrhythmias which may threaten health that are well-known or opaque. The purpose of this pilot study was to examine how different cardiac health risk factors rank together in association with arrhythmias in young, middle-aged, and older adults.

**Methods:**

The analytic sample included 101 adults aged 50.6 ± 22.6 years. Several prominent heart-health-related risk factors were self-reported. Mean arterial pressure and body mass index were collected using standard procedures. Hydraulic handgrip dynamometry measured strength capacity. A 6 min single-lead electrocardiogram evaluated arrhythmias. Respiratory sinus arrhythmias (RSAs) and ectopic heart beats were observed and specified for analyses. Classification and Regression Tree analyses were employed.

**Results:**

A mean arterial pressure ≥ 104 mmHg was the first level predictor for ectopic beats, while age ≥ 41 years was the first level predictor for RSAs. Age, heart rate, stress and anxiety, and physical activity emerged as important variables for ectopic beats (*p* < 0.05), whereas age, sodium, heart rate, and gender were important for RSAs (*p* < 0.05).

**Conclusions:**

RSAs and ectopic arrhythmias may have unique modifiable and non-modifiable factors that may help in understanding their etiology for prevention and treatment as appropriate across the lifespan.

## 1. Introduction

Unlike metronomes that beat with precise, regular, adjustable rhythms, the human heart is very complicated, and its rate of beating is inherently variable. Heart rate (HR) is controlled by a complex host of neural and endocrine factors in response to physical, physiological, and even psychological inputs [1–6]. Some cardiac variations, however, go beyond the ranges of what is considered normal or expected and are regarded as irregularities or arrhythmias. Several types of arrhythmias exist, and whether an arrhythmia is considered benign or serious can be unclear. At least one identified arrhythmia, phasic or respiratory sinus arrhythmia (RSA), is generally considered an arrhythmia with good indication of cardiac response flexibility. Alternatively, some arrhythmias such as ventricular fibrillation, torsade de pointes, and sinus arrests are associated with high mortality risk [7–9]. Most arrhythmias are often moderate or variable in health-related seriousness, with their clinical significance depending on the frequency of occurrence [10]. For example, a well-known arrhythmia, atrial fibrillation (afib), may lead to blood clots that cause strokes and heart attacks, but persons with persistent or even permanent afib can live with these types of arrhythmia for many years, albeit with higher risks of other health problems [11].

Many arrhythmias are infrequently revealed in the general population, with several relatively unknown factors that may contribute to cardiac health. For example, RSAs decline with age and when chronic cardiometabolic disease is present [12]. Similarly, ectopic beats are associated with age, largely asymptomatic, and commonly observed in clinical practice but may signify significant heart disease [13]. Dietary intake such as spicy food, sodium, red meat, and high caffeine consumption are associated with an increased cardiovascular disease risk profile [14–17]. Nicotine is a stimulant, and its use could be a cardiovascular disease risk factor [18]. Anxiety is recognized as a risk factor for poor cardiac health [19]. Hemodynamic stressors from scuba diving may harm cardiovascular functioning [20], while sedentary behavior [21], obesity [22], abnormal blood pressure [23], and low muscle strength [24] are associated with cardiovascular conditions. Accordingly, uncovering how cardiovascular disease risk factors may be linked to arrhythmias such as RSA and ectopic beats may help to guide any appropriate primary and secondary interventions for cardiac health. The purpose of this pilot study was to use consistent methods to evaluate occurrences of arrhythmias and examine how different cardiac health risk factors may be associated with such arrhythmias in young, middle-aged, and older adults.

## 2. Materials and Methods

### 2.1. Participants

**Figure 1.**
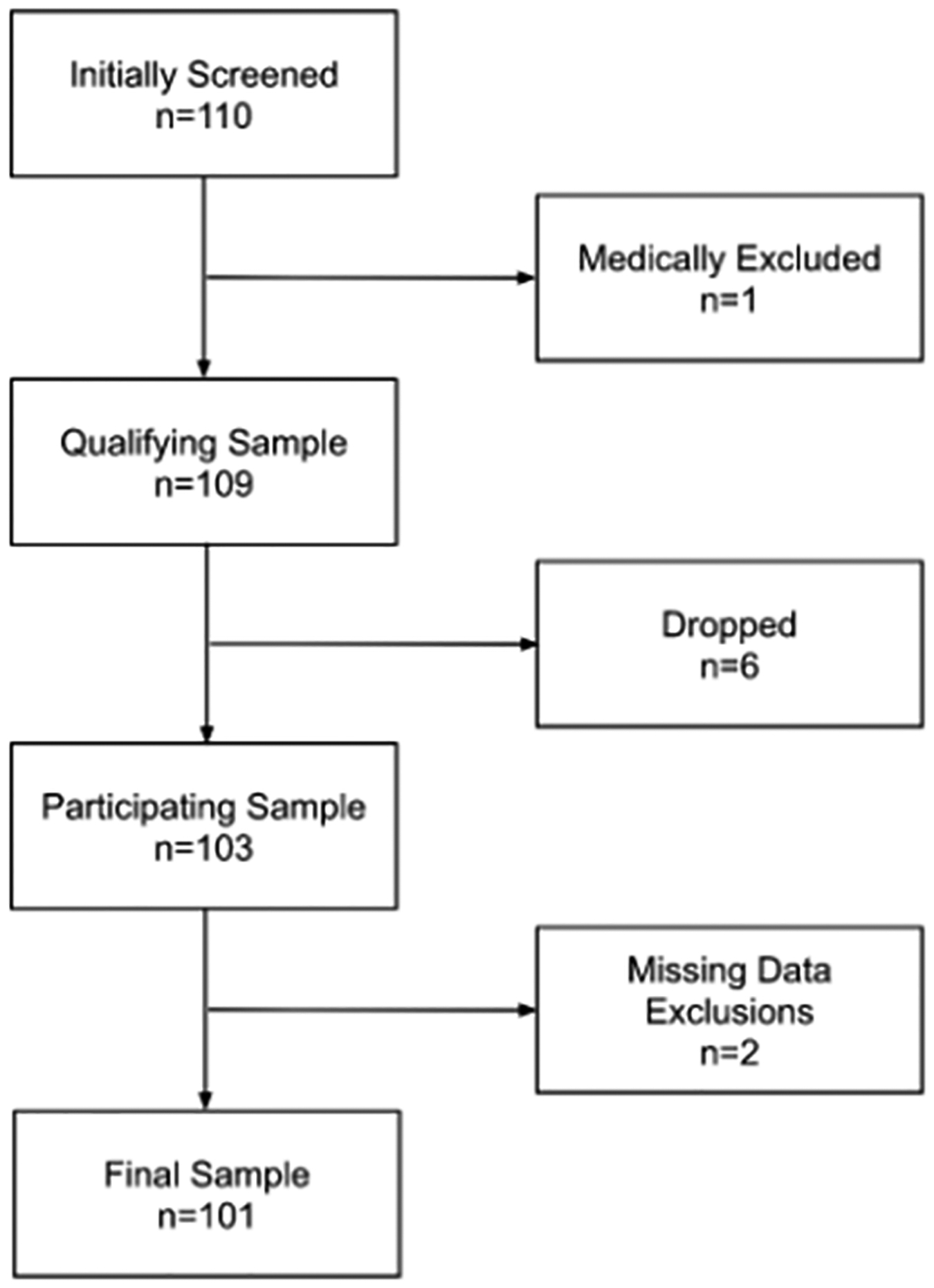
Data flow diagram.

A cross-sectional design was used for this investigation. Generally healthy adult participants were recruited by word-of-mouth, institutionally approved registries, email, and flyers. This investigation was created to examine different types of possible heart health risk factors. Accordingly, we pre-specified the recruitment of approximately 30 participants for detecting statistical power as recommended for pilot studies [25,26]. In order to qualify for this pilot study, participants had to be aged at least 18 years, not currently taking any medication for heart arrhythmias (i.e., only at the time the questionnaire was completed), not have a pacemaker or surgical implant that influences cardiac function, never have had a surgical procedure that influenced cardiac function, and be without severe pain, arthritis, inflammation, or a surgical procedure that would influence heart and handgrip function. Known symptoms of any arrhythmia were not part of screening. A pre-consent screening questionnaire was completed with a trained interviewer to determine eligibility. Of the 110 participants screened at pre-consent; 1 was medically excluded, 6 dropped the study after screening, and 2 were excluded for missing data (Figure 1). Written informed consent was provided by all participants, and the North Dakota State University Institutional Review Board approved the study protocol.

### 2.2. Measures

#### 2.2.1. Self-Report Questionnaire

Each participant was asked questions relating to their demographics, including age, gender, race, marital status, educational achievement, and self-rated health. Dichotomous response items regarding health-related behaviors that are linked to heart function such as spicy food intake (“do you consume food that is considered spicy at least once a week?”), sodium intake (“on a daily basis, do you consume >100% daily value of sodium (2300 mg/day)?”), meat intake (“do you consume red or processed meat more than once/week”), caffeine intake (“do you regularly consume >400 mg of caffeine/day?”), nicotine usage (“are you a current or previous cigarette smoker, use nicotine replacement products, or engage in vaping?”), sleep (“do you get at least 7 h/day of sleep”), stress and anxiety (“would you consider yourself as an anxious or high stress person?”), general physical activity (“Do you regularly engage in 150 min or more of moderate physical activity per week?”), and aquatic activities (“do you currently or in the past 12 months engage in aquatic activities such as swimming, scuba diving, etc.?”). The full questionnaire is included in Supplementary File S1.

#### 2.2.2. Blood Pressure

Participants were asked to sit with feet flat on the floor, with their back against the chair and one arm resting on the table. A trained interviewer placed a cuff around the participant’s upper arm to measure heart rate and oscillometric blood pressure once using an Omron 5 Series Upper Arm Blood Pressure Monitor (Omron; Osaka, Japan). Recorded systolic and diastolic blood pressure were used to calculate mean arterial pressure (MAP) with the following equation: MAP = diastolic pressure + 1/3(systolic pressure-diastolic pressure) [27].

#### 2.2.3. Electrocardiogram

Participants moved to a supine position, and heart rhythm was collected with a 1-lead Heal Force PC–80B electrocardiogram (ECG) using three lead wires and adhesive electrodes (Heal Force Bio-meditech Holdings Group, Shenzhen, China, and Hamburg, Germany). Participants were then instructed to be still, not talk, and keep their eyes closed during the ECG measurement. A trained interviewer recorded the time the ECG started for a 6 min duration for obtaining at least a conventional 240–300 s recording [28,29]. Upon completion of the 6 min recording, the electrodes were removed, and the ECG recording was uploaded to the ECG device’s software for processing (ECG Data Manager V5.2.01).

After each visit, a trained interviewer analyzed the ECG and completed a cover sheet. The minimum, maximum, and average HR were recorded from the software’s automatically measured algorithm and visually confirmed by a trained interviewer for atypical departures of HR. Each recording was carefully inspected for RSAs. An electronic caliper in the device’s software was used to determine the maximum difference among inter-beat intervals by R wave to R wave (RR), in milliseconds, between beats of representative, estimated normal respiratory periods. We considered the commonly used time of 120 ms as the criterion for RSA. Any ectopic/premature junctional complex (PJC), premature atrial complex (PAC), or premature ventricular complex (PVC) were noted from the recording along with any unknown ectopic beats. The reading was further checked for other arrhythmias including afib, atrial flutter (aflutter), supra ventricular tachycardia (SVT), and AV blocks (which are associated with >240 ms PR interval and dropped QRS, or disassociated P and QRS, and sinus pauses which are associated with RR > 2 s). Potential non-arrhythmia issues that were monitored included PR interval <120 ms and delta wave, ST elevation and depression >3 mm, long QTc interval, and inverted or other abnormal T waves.

We initially sought to include any known arrhythmia. However, we observed no afib or other major, clinically important arrhythmias, with sufficient numbers for statistical analyses of only RSAs and ectopic beats.

#### 2.2.4. Physical Measures

All physical measures were collected after the ECG recording to avoid impacting blood pressure, HR, and arrhythmias. Standing height was measured to the nearest 0.5 cm and body mass to the nearest 0.1 kg with the Seca 286 measuring station (Seca; Chino, CA, USA). Body mass index was calculated as kg/m^2^. Handgrip strength was measured with a Jamar hydraulic handgrip dynamometer (Lafayette Instrument Company; Lafayette, IN, USA) and Biopac SS25LA electronic handgrip dynamometer (Biopac Systems; Goleta, CA, USA). Participants were asked to sit comfortably with their forearms on the arms of the chair, wrist in a neutral position just over the end of the arm of the chair, and thumb facing upwards. A trained interviewer explained the handgrip strength protocol and participants were allowed a practice trial with each dynamometer after the dynamometer was fitted to the hand size of each participant as appropriate. Start hand and dynamometer first used was block randomized. Then, participants squeezed the dynamometer with maximal effort for 3 trials on each hand, alternating between hands. A 1 min rest period was provided between measures. Males and females with handgrip strength <35.5 kg and <20 kg were considered weak, respectively [30,31]. Participants completed the handgrip strength protocol in full with one dynamometer before reengaging in the protocol with the other dynamometer. The highest recorded handgrip strength from the hydraulic dynamometer was included in the primary and supplementary analyses, while the highest recorded handgrip strength from the electronic dynamometer was only used in the supplementary analysis.

### 2.3. Statistical Analysis

Analyses were performed with SAS 9.4 software (SAS Institute; Cary, NC, USA), its menu-driven siblings JMP 15 and 17, or R-Studio (Posit; Boston, MA, USA). Included measures were standardized to have a mean of 0 and variance of 1 because they were measured on different scales. Thereafter, Pearson correlations quantified the relationships between age, gender, spicy food intake, sodium intake, caffeine intake, processed meat intake, nicotine intake, sleep, physical activity participation, self-rated health, aquatic activities, stress and anxiety, body mass index, handgrip strength, MAP, HR, ectopic arrhythmias, and RSA. Qualitative interpretation terms (medical) were utilized to describe the strength of the correlation coefficients [32].

We performed a principal component analysis with varimax rotation to derive a collection of new uncorrelated variables (i.e., principal components) for age, gender, spicy food intake, sodium intake, caffeine intake, processed meat intake, nicotine intake, sleep, physical activity participation, self-rated health, aquatic activity participation, stress and anxiety, body mass index, handgrip strength, mean arterial pressure, mean heart rate, ectopic arrhythmias, and RSA. Overall, the purpose of the principal component analysis is to decompress a collection of related variables into another type of uncorrelated variables that are displayed in descending order of variance as a type of data reduction technique [33]. This linear combination represents the dimension along which the measures are maximally separated. In agreement with the Kaiser–Guttman criteria, principal components with eigenvalues >1.0 were retained [34]. Factor loadings |>0.50| within each principal component were similarly retained [35].

Following the principal component analysis, we further examined some of the primary single factors of interest with standard bivariate (Y by X) analyses depending on whether the data were continuous, ordinal, or nominal, hence, linear or logistic regression, ANOVA, or contingency tables along with their appropriate measures (e.g., F values or chi-square) for determining *p*-values for null hypothesis testing. Because we aggregated some of the continuous data into categories such as age into three groups, RR variation into RSA presence or absence, and ectopic counts into presence or absence, and since inferences can depend and vary due to aggregation [36], we double-checked the resulting inferential outcomes by analyzing both the continuous and aggregated data for comparison.

Classification and Regression Tree (CART) analyses were utilized for the predictive association of age, gender, spicy food intake, sodium intake, caffeine intake, processed meat intake, nicotine intake, sleep, physical activity participation, self-rated health, aquatic activities, stress and anxiety, body mass index, handgrip strength, MAP, and HR on (1) ectopic beats, and (2) RSAs. This method of supervised machine learning yielded results that were presented as decision trees with relevant coverage for each classification in the prediction algorithms and for overall importance of each explanatory variable included. An alpha level of 0.05 was used for relevant analyses.

As a supplementary analysis, we examined the relationships between the hydraulic- and electronic-dynamometer-derived handgrip strength measurements using an interclass correlation coefficient. Interpretation of such coefficients was used (<0.50 is poor; 0.50–0.75 is moderate; 0.76–0.90 is good; >0.90 is excellent) [37]. According to the National Heart, Lung, and Blood Institute, weakness is a symptom of heart arrhythmias [38], and given that persons are often not showing this symptom (i.e., asymptomatic), another supplementary analysis was performed using the same statistical procedures from our principal component analyses by weakness status.

## 3. Results

### 3.1. Descriptive Characteristics

The descriptive characteristics of the participants are presented in Table 1. Overall, participants were aged 50.6 ± 22.6 years and were mostly female (60.0%). Of the 101 participants in our study, 34 (33.7%) were aged 18–44 years, 26 (25.7%) were aged 45–64 years, and 41 (40.6%) were aged ≥65 years.

### 3.2. Arrhythmias and Other Cardiac Conditions

**Figure 2.**
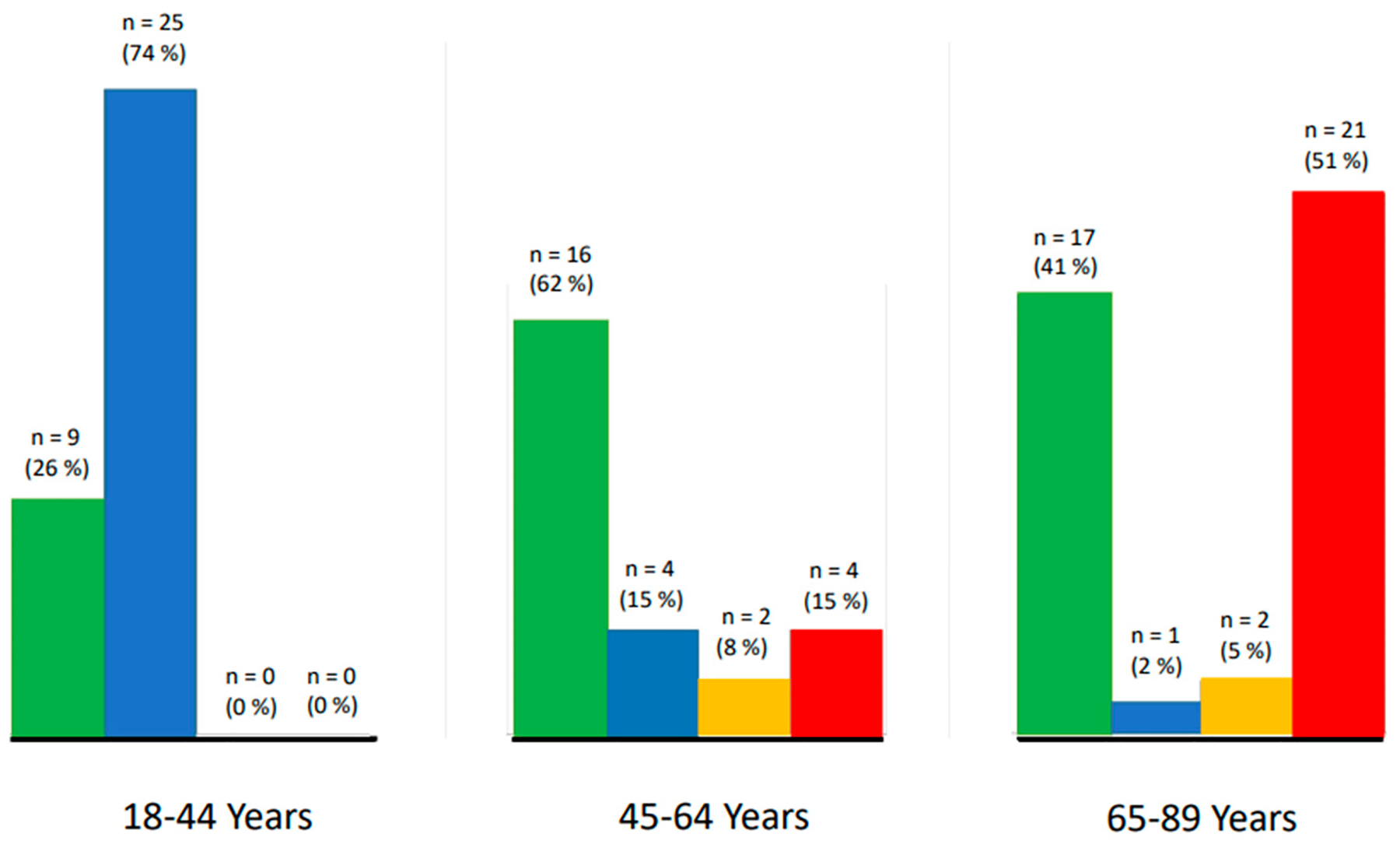
Arrhythmia by age category. Note: green = neither respiratory sinus arrhythmia or ectopic; blue = respiratory sinus arrhythmia only; yellow = respiratory sinus arrhythmia and ectopic; red = ectopic only.

RSA frequency was predominant in the young adult group and trailed off (but was still present) in the other two age groups, whereas ectopic beats were absent in our samples from the young adults but increasingly emerged in the middle and older ages (Figure 2).

### 3.3. Factors Possibly Associated with Arrhythmias

Supplementary Table S1 presents the correlation matrix of the standardized measures. Age (r = −0.61, *p* < 0.01), stress and anxiety (r = 0.24, *p* = 0.01), body mass index (r = −0.31, *p* < 0.01), handgrip strength (r = 0.20, *p* = 0.04), MAP (r = −0.34, *p* < 0.01), and mean heart rate (r = −0.25, *p* < 0.01) were correlated with RSAs.

**Figure 3.**
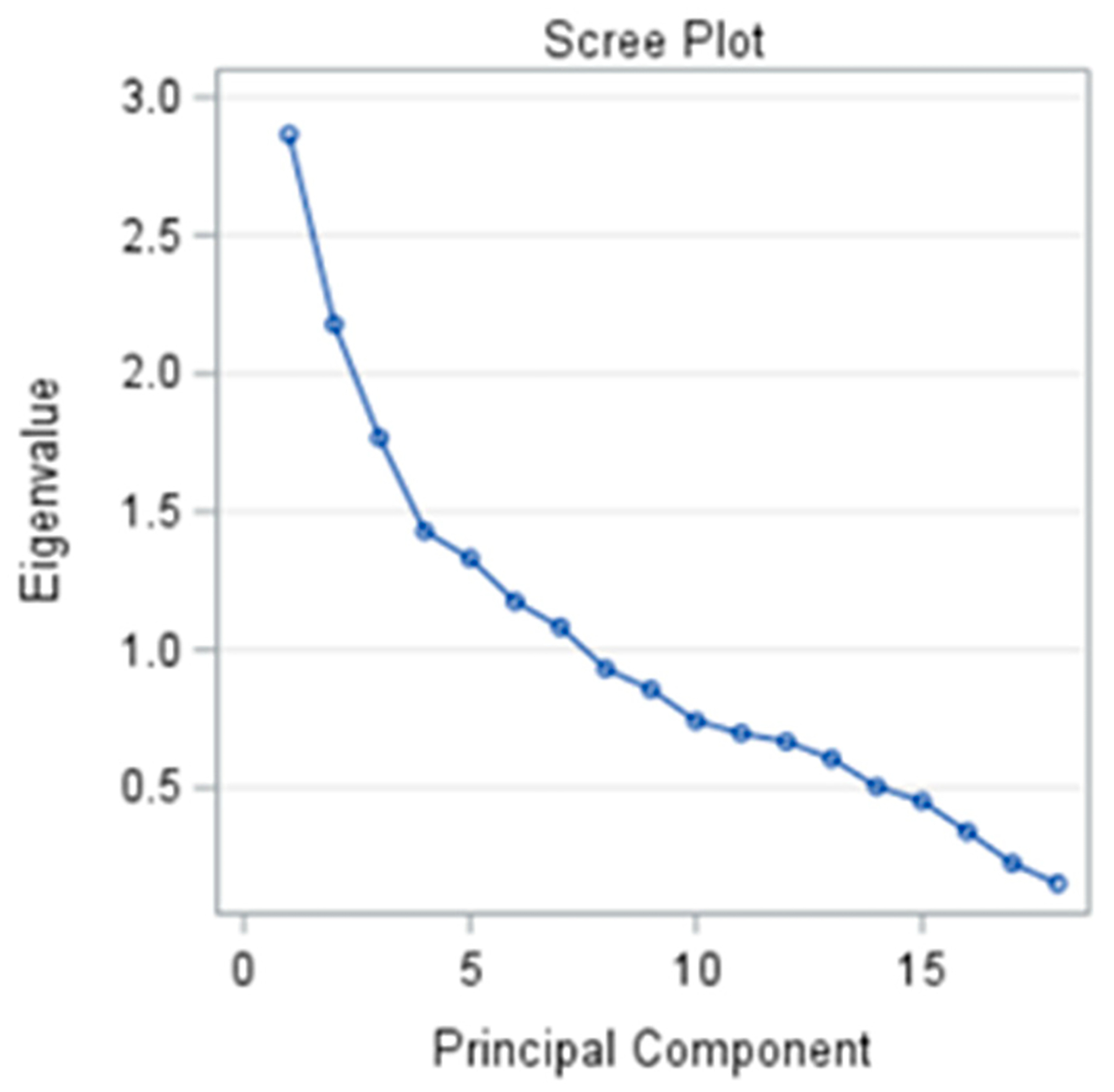
Principal component scree plot.

Figure 3 shows a scree plot for the principal components. We retained principal components 1, 2, 3, 4, 5, 6, and 7 because their eigenvalues were 2.87, 2.23, 1.75, 1.41, 1.32, 1.15, and 1.08, respectively. Table 2 presents the factor loading for the principal component analysis. Age, stress and anxiety, body mass index, MAP, and RSA loaded to the first principal component, which explained 20.3% of the variance. Principal component 7, which included 10.6% of the variance, included total ectopic with sodium intake. All other potential factors that we considered were not connected with significant loading with either RSA or ectopic heart beats.

### 3.4. Respiratory Sinus Arrhythmia

**Figure 4.**
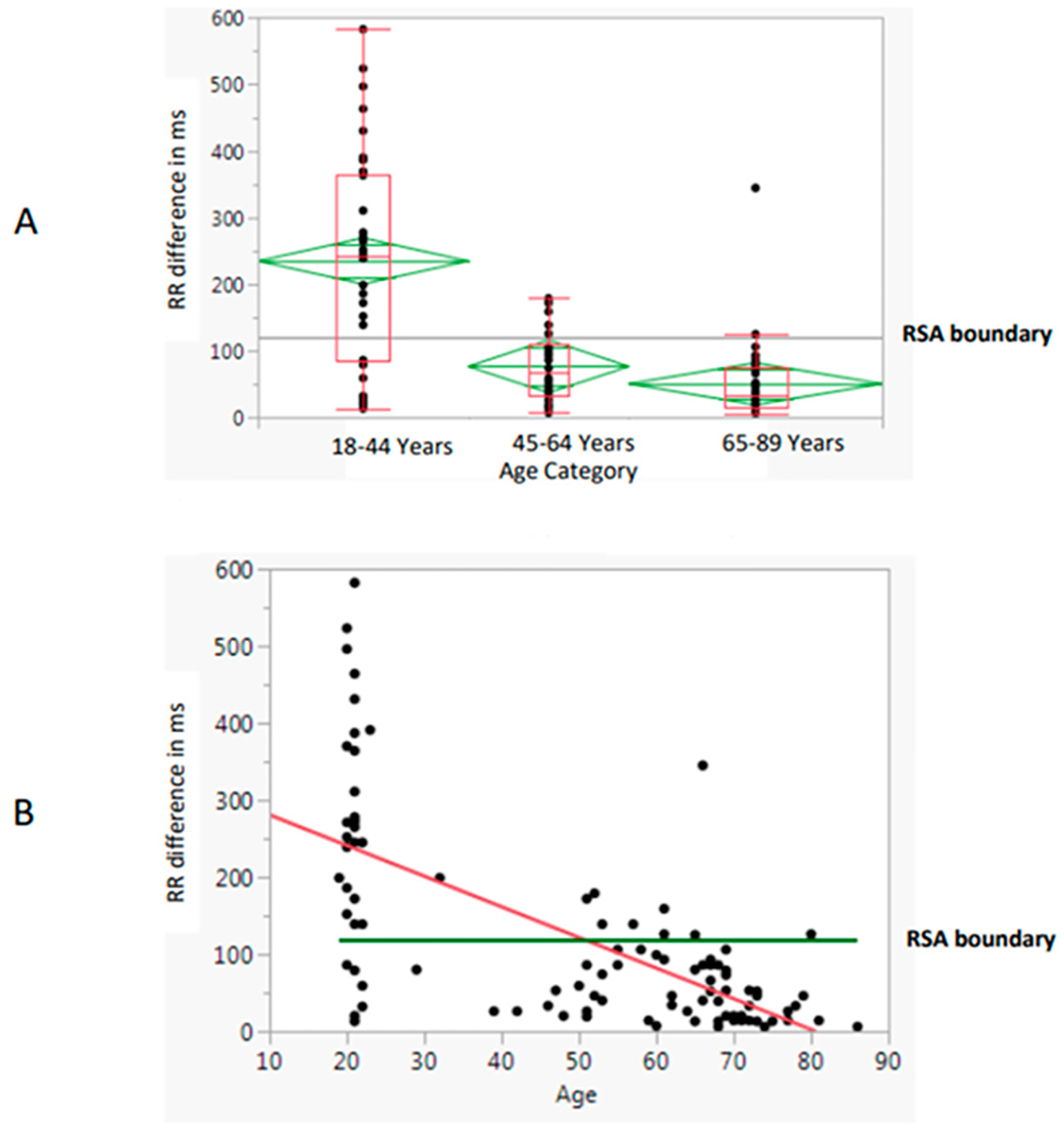
RR Difference by (**A**) age category and (**B**) age. Note: RSA = respiratory sinus arrhythmia.

**Figure 5.**
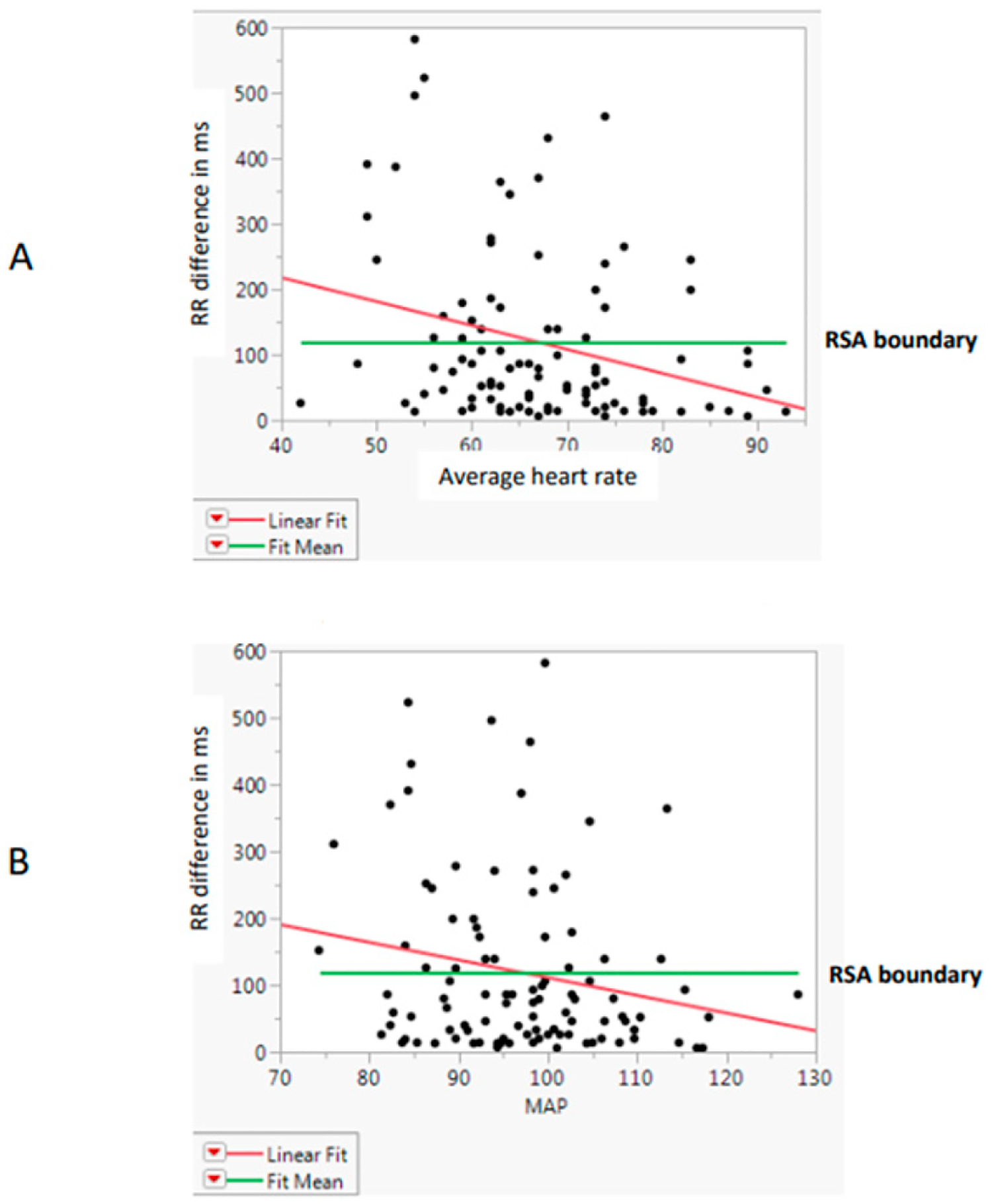
RR difference relative to (**A**) average heart rate and (**B**) mean arterial pressure. Note: MAP = mean arterial pressure; RSA = respiratory sinus arrhythmia.

The highly significant relationship with RR differences (*p* < 0.01) emerged by age category (Figure 4A) and by actual ages (Figure 4B). RR differences overall were also analyzed by linear fit and ANOVA for two other heart-related variables, average HR and MAP. They were highly significant (F = 8.59, *p* = 0.0042) for HR, with the RR difference declining with the increase in HR (Figure 5A) but being barely significant (F = 4.10, *p* = 0.046) for MAP (Figure 5B).

### 3.5. Ectopic Arrhythmias

The age relationship for ectopic arrhythmias was further examined not just by the significant relationship with the age category but also by actual age. Interestingly, the relationship when using total ectopic counts vs. actual age still showed the trend, but it was not statistically significant (ANOVA, F = 3.12, *p* = 0.08).

We examined the role of PVCs compared to non-PVC ectopic beats. Regarding the prevalence of PVC presence vs. non-PVC ectopic beat presence, there were 9 cases with PVCs only, 17 cases with non-PVC ectopics only, 3 cases with both, 72 cases with neither, and no significant difference in their frequencies (contingency test LogLike R square = nearly zero, Likelihood Ratio and Pearson chi-square = 0.22, *p* > 0.63).

### 3.6. Supervised Machine Learning

**Figure 6.**
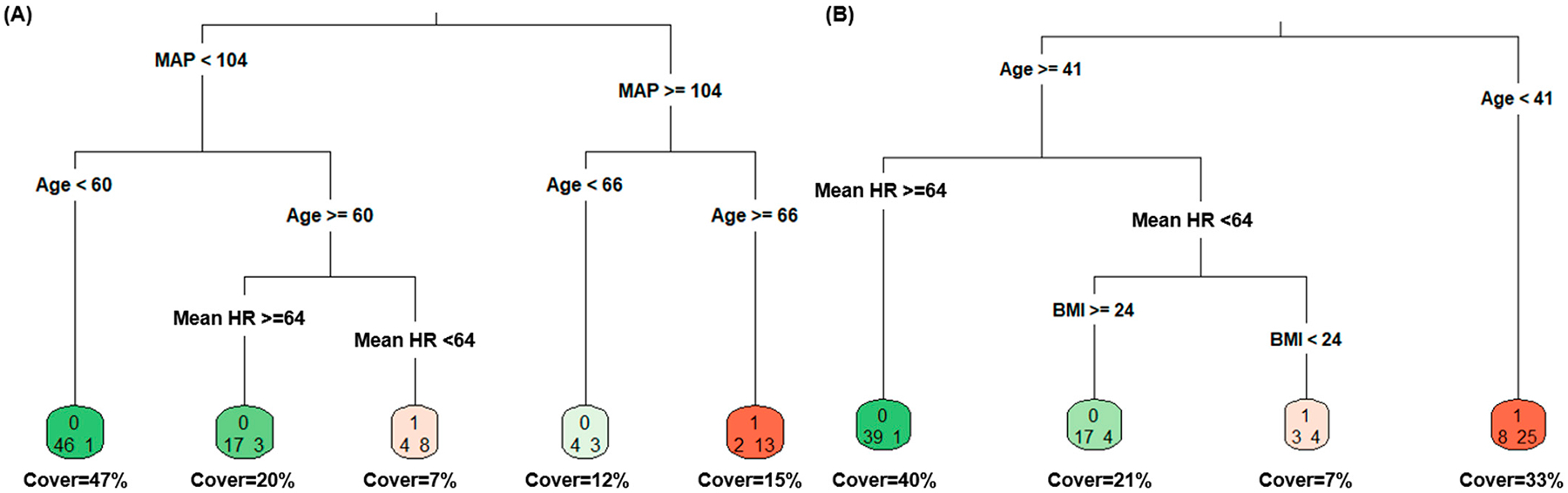
Decision trees for (**A**) ectopic beats and (**B**) respiratory sinus arrhythmias. Note: BMI = body mass index; HR = heart rate.

**Figure 7.**
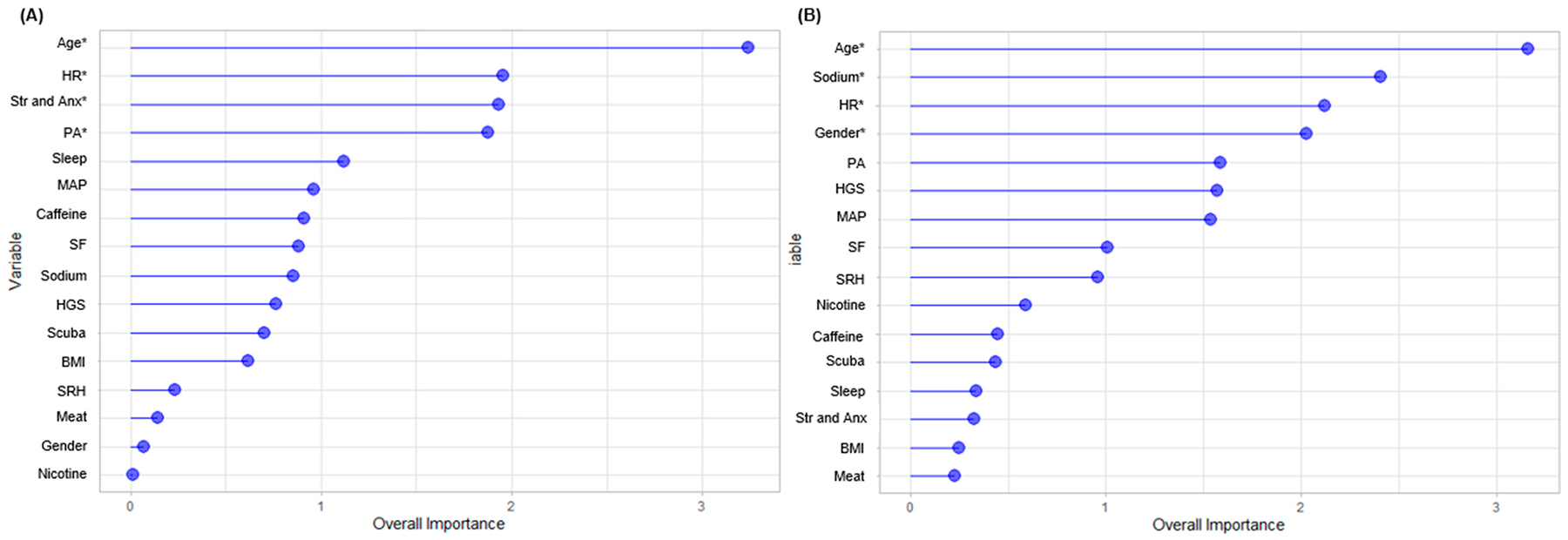
Variable importance for (**A**) ectopic beats and (**B**) respiratory sinus arrythmias. Note: * *p* < 0.05. BMI = body mass index; HGS = handgrip strength; HR = heart rate; MAP = mean arterial pressure; PA = physical activity; SF = spicy food intake; SRH = self-rated health; Str and Anx = stress and anxiety.

Figure 6 presents the decision trees for ectopic beats and RSAs. For ectopic beats, a MAP ≥ 104 mmHg was the first level predictor, with age and HR being factors for subsequent splits in the tree. RSAs included age at ≥41 years as the first level predictor, with divergence thereafter which included HR and BMI. Figure 7 displays the overall variable importance for ectopic beats and RSAs. Age, mean HR, stress and anxiety, and physical activity were revealed as significant factors for variable importance with ectopic beats (*p* < 0.05), while age, sodium intake, mean HR, and gender were relevant for RSAs (*p* < 0.05).

### 3.7. Supplementary Analysis

The interclass correlation coefficient analysis revealed that handgrip strength collected from the hydraulic and electronic dynamometers was excellent and positive (0.94). Supplementary Table S2 shows the factor loading for the principal component analysis in participants that were considered weak. Ectopic beats loaded with MAP, stress and anxiety, scuba diving, sodium intake, and age in principal component 2, which explained 20.5% of the variance. However, ectopic beats also loaded with physical activity, sleep, and caffeine intake in principal component 5, which explained 17.5% of the variance. The factor loading for the principal component analysis in participants that were not weak is presented in Supplementary Table S3. RSAs loaded with age, body mass index, and stress and anxiety in principal component 2, which accounted for 19.7% of the variance. Ectopic beats loaded with sodium intake in principal component 6, which accounted for 10.9% of the variance.

## 4. Discussion

The overarching findings from this pilot investigation suggest that age, stress, body mass index, and MAP loaded with RSAs. Likewise, sodium intake loaded with ectopic arrythmias. Age was also influential with respect to RSA and ectopic arrythmias, such that RSA was more pronounced at younger ages, while ectopic arrythmias were more frequently observed in older adults. There were several cardiac risk factors that did not load with RSA and ectopic beats, signifying the possible importance of certain factors in their relationship with arrythmias than others. Our decision tree presented preliminary clinical utility for prediction of ectopic beats and RSAs. Age, HR, stress and anxiety, and physical activity could be important for predicting ectopic beats, while age, sodium intake, HR, and gender might be important for RSAs. These pilot findings underscore the role of certain risk factors for arrythmia type, thereby serving as possible targets for mitigating arrythmias that may influence health.

Other factors may serve as targets for non-RSAs aside from age. Given that we observed RSAs more frequently in younger adults, stress mitigation strategies for non-RSAs should be considered in younger populations such as college students for autonomic nervous system health [39]. Adiposity related to body mass index may lead to the inflammation and epicardial fats that drive the fatty infiltration lipotocicity connected to atrial and ventricular arrhythmias [40]. Arterial blood pressure may serve as an RSA stabilization mechanism [41]. Accordingly, stress, body mass index, and MAP could be useful targets for explaining RSAs.

This pilot investigation also found that sodium intake loaded with ectopic beats. Sodium intake is a known predictor of the increased blood pressures that lead to heart disease [42]. Interestingly, sodium may likewise be connected with atrial tachycardia apart from hypertension due to elevated intracellular calcium levels transitioning in the exchange mechanisms of cardiac tissues, which in turn influences the release of intracellular calcium from the sarcoplasmic reticulum leading to arrhythmias [43]. Adults may consider maintaining adherence to dietary recommendations for daily sodium intake in an effort to mitigate ectopic arrythmias [42].

Handgrip strength is associated with cardiovascular disease and cardiovascular disease mortality [44] and has been regarded as a stronger predictor of all-cause and cardiovascular mortality than systolic blood pressure [24]. Given that handgrip dynamometer technologies are evolving beyond conventional hydraulic dynamometers [45], comparing existing new and conventional dynamometers will help in adoption of such dynamometer technologies. The findings from our supplementary analysis suggest an excellent interclass correlation coefficient between handgrip strength measured with the hydraulic and electronic dynamometers. As the clinical utilization of handgrip strength increases alongside improvements in equipment, our findings indicate that electronic handgrip dynamometers are viable tools for collecting strength capacity. Moreover, the findings from our weakness stratified principal component analyses are somewhat parallel to those from our primary analyses. For example, ectopic beats loaded with sodium intake in persons that were not weak, whereas ectopic beats similarly loaded with sodium intake in persons with weakness. Such findings underscore the role of weakness as a symptom of cardiac arrhythmias.

Apart from the findings from our principal component analyses, the CART analyses provided decision trees that may help in the prediction of ectopic beats and RSAs. Likewise, age, HR, stress and anxiety, and physical activity were identified as important for ectopic beats, while age, sodium intake, HR, and gender could be important for RSAs. While there are a multitude of risk factors for different heart arrhythmias, these pilot findings provide initial clinical utility for determining ectopic beats and RSAs and may serve as targets for interventions devoted to heart health. Applying CART analyses to larger datasets (e.g., UK Biobank) may help to support or dispute our preliminary findings and should be considered for future research.

Some limitations should be acknowledged. Participants did not report a specific medication-free period for any heart arrhythmia to reduce recall bias and increase their convenience. A single-lead rhythm ECG with 6 min recording time was used in this pilot investigation for feasibility. A longer ECG rhythm recording duration might have improved RSA and ectopic arrythmia detections. Factors such as breathing patterns and ranges were not controlled during ECG assessments, although we advised participants to remain relaxed in a supine position. ECG assessments require an intricate evaluation of thousands of heart beats, and while we utilized several quality controls, measurement and misinterpretation errors may have occurred. We utilized simple self-reporting of factors included in our analyses, which introduces biases. Although our sample size may have been adequate for a pilot-level investigation, our sample size and inclusion of generally healthy, community-dwelling adults may have limited generalizability to clinical and morbid populations. Future research should continue examining how different factors may impact arrythmias with different experimental designs. Specifically, research is warranted for asymptomatic arrhythmias, and the emergence of apps and devices that feasibly assess heart health with ECG-related technologies may provide new opportunities for screening; however, comprehensive ECG testing should be performed by a healthcare provider for any diagnostic-related purposes.

## 5. Conclusions

This pilot study found that stress, anxiety, body mass index, and mean arterial pressure loaded with RSAs, and sodium intake loaded with ectopic arrhythmias. Age is also a hallmark in arrhythmia type presence, wherein many younger adults had RSA, while older adults had ectopic beats. The results of our decision trees may serve as clinical tools for detecting ectopic beats and RSAs. Age, HR, stress and anxiety, and physical activity were identified as important for ectopic beats, whereas age, sodium, HR, and gender were important for RSAs. Our findings provide possible targets for primary and secondary intervention with respect to controlling arrhythmia type as applicable. More research on risk factors for arrhythmias with longitudinal designs, asymptomatic cases, and sophisticated ECG technologies are warranted.

## Supplementary Material

Supplementary Material

## Figures and Tables

**Table 1.** Descriptive characteristics of the participants.

Variable	Overall (*n* = 101)	Aged 18–44 Years (*n* = 34)	Aged 45–64 Years (*n* = 26)	Aged 65–89 Years (*n* = 41)
Age (years)	50.6 ± 22.6	22.5 ± 5.1	55.1 ± 5.2	71.0 ± 4.8
Race (*n* (%))				
Asian	2 (2.0)	1 (3.9)	1 (3.9)	0 (0.0)
White	97 (96.0)	32 (92.2)	34 (92.2)	41 (100.0)
More than One Race	2 (2.0)	1 (3.9)	1 (3.9)	0 (0.0)
Marital Status (*n* (%))				
Single	46 (45.5)	31 (91.2)	4 (15.4)	11 (26.8)
Married	44 (43.6)	3 (8.8)	22 (84.6)	19 (46.3)
Widowed	10 (9.9)	0 (0.0)	0 (0.0)	10 (24.4)
Other	1 (1.0)	0 (0.0)	0 (0.0)	1 (2.5)
Education (*n* (%))				
High School Graduate/GED	12 (11.9)	9 (26.5)	0 (0.0)	3 (7.3)
Some College	32 (31.7)	18 (53.0)	4 (15.4)	10 (24.4)
Associate’s Degree	7 (6.9)	1 (2.9)	2 (7.7)	4 (9.8)
Bachelor’s Degree	32 (31.7)	3 (8.8)	15 (57.7)	14 (34.1)
Graduate Degree	18 (17.8)	3 (8.8)	5 (19.2)	10 (24.4)
Hydraulic Handgrip Strength (kg)	35.5 ± 12.1	42.2 ± 10.7	36.8 ± 10.8	29.1 ± 10.8
Weakness (*n* (%))	7 (12.8)	0 (0.0)	0 (0.0)	7 (17.1)
Body Mass Index (kg/m^2^)	28.1 ± 5.7	25.0 ± 5.1	30.1 ± 6.4	29.4 ± 6.0
Mean Arterial Pressure (mmHg)	96.9 ± 9.8	92.8 ± 8.6	97.6 ± 8.8	99.8 ± 10.0
Mean Heart Rate (beats/minute)	66.9 ± 10.1	66.0 ± 10.2	66.7 ± 8.8	67.9 ± 11.0
Female (*n* (%))	61 (60.0)	16 (47.1)	18 (69.2)	27 (65.9)
Spicy Food Intake (*n* (%))	53 (52.5)	18 (52.9)	15 (57.6)	20 (48.8)
Sodium Intake (*n* (%))	46 (45.5)	20 (58.8)	11 (42.3)	15 (36.6)
Caffeine Intake (*n* (%))	30 (29.7)	6 (17.6)	9 (34.6)	15 (36.6)
Meat Intake (score)	1.9 ± 0.7	2.2 ± 0.8	1.8 ± 0.7	1.8 ± 0.7
Uses Nicotine (*n* (%))	9 (8.9)	3 (8.8)	2 (7.6)	4 (9.8)
<7 h of Sleep (*n* (%))	29 (28.7)	7 (20.5)	2 (7.7)	15 (36.6)
Self-Rated Health (*n* (%))				
Excellent	14 (13.9)	4 (11.8)	4 (15.4)	6 (14.6)
Very Good	48 (47.5)	18 (52.9)	11 (42.3)	19 (46.4)
Good	36 (35.6)	11 (32.4)	9 (34.6)	16 (39.0)
Fair	3 (3.0)	1 (2.9)	2 (7.7)	0 (0.0)
Poor	0 (0.0)	0 (0.0)	0 (0.0)	0 (0.0)
Scuba Diving Participation (score)	0.4 ± 0.7	0.5 ± 0.8	0.6 ± 0.7	0.3 ± 0.5
Stress and Anxiety (score)	0.5 ± 0.8	0.8 ± 0.9	0.5 ± 1.6	0.3 ± 0.6

Note: Results are presented as mean ± standard deviation or frequency (percentage) as indicated.

**Table 2.** Factor loadings for the principal component analysis.

Variable	PC1	PC2	PC3	PC4	PC5	PC6	PC7
Age	0.78 *	−0.36	−0.02	0.05	0.04	−0.07	0.23
Gender	−0.12	−0.88 *	0.02	−0.03	0.01	−0.12	0.02
Spicy Food Intake	−0.04	0.33	−0.15	0.03	0.57 *	−0.27	0.13
Sodium Intake	−0.01	0.23	−0.02	−0.31	0.13	−0.18	−0.71 *
Caffeine Intake	0.16	0.13	0.40	0.46	0.10	−0.22	0.02
Processed Meat Intake	−0.20	0.36	0.28	−0.53 *	0.22	0.10	−0.09
Nicotine Intake	0.05	0.02	0.77	−0.11	−0.12	0.06	−0.09
Sleep	0.16	−0.13	0.08	−0.01	0.77 *	0.09	−0.10
Physical Activity Participation	−0.01	−0.27	0.71	0.24	0.19	−0.02	0.13
Self-Rated Health	0.06	0.09	0.08	0.74 *	0.03	0.16	−0.07
Scuba Diving Participation	−0.03	0.25	−0.03	0.08	−0.06	0.70 *	0.07
Stress and Anxiety	−0.59 *	−0.14	−0.07	0.36	0.09	−0.35	0.07
Body Mass Index	0.55 *	0.10	0.06	0.27	0.20	0.01	−0.04
Handgrip Strength	−0.16	0.89 *	−0.05	0.01	−0.01	0.06	−0.01
Mean Arterial Pressure	0.52 *	0.25	−0.19	−0.03	−0.25	−0.43	0.20
Mean Heart Rate	0.25	0.10	0.43	0.07	−0.36	−0.41	−0.10
Total Ectopic	0.14	0.15	−0.04	−0.33	0.09	−0.09	0.73 *
Respiratory Sinus Arrhythmia	−0.78 *	0.07	−0.12	−0.11	−0.03	0.12	−0.01
Variance Explained	20.3%	19.8%	14.0%	13.4%	11.2%	10.6%	10.6%

* Significant factor loading (|>0.50|). Note: PC = principal component.

## Data Availability

The data presented in this study are available in this article (and Supplementary Materials).
