# Supplementary material for: Supervised Machine Learning to Examine Factors Associated with Respiratory Sinus Arrhythmias and Ectopic Heart Beats in Adults: A Pilot Study
Source: Hearts (Basel). Author manuscript; Available in PMC 2025 Jan 31. (PMC11784985; doi:10.3390/hearts5030020)
Supplement: Supplementary Material [file NIHMS2011978-supplement-Supplementary_Material.pdf]

Supplementary Table S1. Correlation Matric of the Standardized Measures.

	Age	Gender	Spicy Food	Sodium	Caffeine	Processed Meat	Nicotine	Sleep	Physical Activity
**Age**	-	r=0.18; p=0.06	r=-0.05; p=0.61	r=-0.22; p=0.02	r=0.17; p=0.07	r=-0.34; p<0.01	r=-0.01; p=0.97	r=0.11; p=0.24	r=0.12; p=0.21
**Gender**		-	r=-0.16; p=0.10	r=-0.15; p=0.12	r=-0.09; p=0.35	r=-0.25; p<0.01	r=-0.03; p=0.75	r=0.02; p=0.82	r=0.23; p=0.01
**Spicy Food**			-	r=0.07; p=0.46	r=0.01; p=0.91	r=0.07; p=0.43	r=-0.11; p=0.23	r=0.16; p=0.09	r=-0.09; p=0.32
**Sodium**				-	r=-0.02; p=0.77	r=0.22; p=0.02	r=0.06; p=0.53	r=0.03; p=0.72	r=-0.18; p=0.06
**Caffeine**					-	r=-0.15; p=0.12	r=0.17; p=0.07	r=0.11; p=0.25	r=0.29; p<0.01
**Processed Meat**						-	r=0.09; p=0.32	r=0.07; p=0.46	r=0.01; p=0.90
**Nicotine**							-	r=0.03 p=0.75	r=0.29; p<0.01
**Sleep**								-	r=0.14; p=0.15
**Physical Activity**									-
**Self-Rated Health**									
**Scuba Diving**									
**Stress & Anxiety**									
**BMI**									
**HGS**									
**MAP**									
**Mean HR**									
**Total Ectopic**									
**RSA**									

	Self-Rated Health	Scuba Diving	Stress & Anxiety	BMI	HGS	MAP	Mean HR	Total Ectopic	RSA Only
**Age**	r=-0.02; p=0.79	r=-0.13; p=0.17	r=-0.29; p<0.01	r=0.35; p<0.01	r=-0.43; p<0.01	r=0.33; p<0.01	r=0.09; p=0.34	r=0.17; p=0.08	r=-0.61; p<0.01
**Gender**	r=-0.10; p=0.28	r=-0.21; p=0.03	r=0.19; p=0.05	r=-0.06; p=0.52	r=-0.77; p<0.01	r=-0.21; p=0.02	r<-0.01; p=0.94	r=-0.07; p=0.48	r=0.06; p=0.53
**Spicy Food**	r<0.01; p=0.93	r<0.01; p=0.92	r=0.03; p=0.75	r=0.10; p=0.28	r=0.19; p=0.04	r=-0.01; p=0.92	r=0.02; p=0.81	r=0.13; p=0.18	r=0.04; p=0.62
**Sodium**	r=-0.15; p=0.11	r=-0.03; p=0.69	r=-0.03; p=0.75	r=-0.01; p=0.90	r=0.20; p=0.04	r=-0.09; p=0.36	r=0.04; p=0.68	r=-0.13; p=0.17	r=-0.02; p=0.83
**Caffeine**	r=0.16; p=0.09	r=-0.02; p=0.77	r=0.05; p=0.60	r=0.13; p=0.17	r=0.04; p=0.62	r=0.10; p=0.28	r=0.17; p=0.07	r=-0.08; p=0.40	r=-0.18; p=0.05
**Processed Meat**	r=-0.18; p=0.06	r=0.05; p=0.59	r=-0.11; p=0.24	r=-0.07; p=0.47	r=0.27; p=0.01	r=-0.09; p=0.33	r=-0.03; p=0.75	r=0.06; p=0.49	r=0.18; p=0.06
**Nicotine**	r=0.02; p=0.81	r<-0.01 p=0.92	r=-0.11; p=0.25	r=0.02; p=0.79	r=-0.03; p=0.73	r=-0.09; p=0.35	r=0.27; p<0.01	r=-0.04; p=0.62	r=-0.07; p=0.45
**Sleep**	r=0.02 p=0.77	r=-0.01; p=0.87	r=-0.01; p=0.85	r=0.08; p=0.37	r=-0.13; p=0.18	r=-0.05; p=0.55	r=-0.16; p=0.09	r=-0.04; p=0.64	r=-0.12; p=0.20
**Physical Activity**	r=0.16; p=0.10	r=-0.13; p=0.17	r=0.08; p=0.42	r=0.13; p=0.19	r=-0.20; p=0.18	r=-0.20; p=0.03	r=0.16; p=0.09	r=-0.07; p=0.42	r=-0.18; p=0.06
**Self-Rated Health**	-	r=0.03; p=0.71	r=0.09; p=0.39	r=0.21; p=0.03	r=0.01; p=0.85	r=-0.07; p=0.44	r=0.06; p=0.54	r=-0.16; p=0.10	r=-0.15; p=0.12
**Scuba Diving**		-	r=-0.06; p=0.50	r<-0.01; p=0.94	r=0.18; p=0.06	r=-0.17; p=0.07	r=-0.07; p=0.46	r=0.01; p=0.93	r=0.09; p=0.35
**Stress & Anxiety**			-	r=-0.16; p=0.09	r=-0.06; p=0.50	r=-0.16; p=0.09	r=-0.07; p=0.43	r=-0.07; p=0.46	r=0.24; p=0.01
**BMI**				-	r=-0.01; p=0.94	r=0.17; p=0.07	r=0.15; p=0.11	r=0.03; p=0.76	r=-0.31; p<0.01
**HGS**					-	r=0.09; p=0.32	r=-0.07; p=0.44	r=0.06; p=0.53	r=0.20; p=0.04

**MAP**	-	r=0.24; p=0.01	r=0.19; p=0.05	r=-0.34; p<0.01
**Mean HR**		-	r=-0.05; p=0.56	r=-0.25; p<0.01
**Total Ectopic**			-	r=-0.13; p=0.18
**RSA**				-

Supplementary Table S2. Factor Loadings for the Principal Component Analysis in Persons with Weakness.

Variable	PC1	PC2	PC3	PC4	PC5
Age	0.02	0.58*	0.78*	-0.15	0.07
Gender	-0.95	0.08	-0.17	-0.08	0.19
Spicy Food Intake	0.30	0.17	0.02	0.88*	-0.26
Sodium Intake	0.27	0.55*	0.01	-0.72*	0.29
Caffeine Intake	0.23	0.06	0.60*	-0.35	0.51*
Processed Meat Intake	0.71*	-0.03	0.35	-0.34	0.32
Nicotine Intake	0.25	-0.02	0.87*	0.36	-0.18
Sleep	0.02	-0.19	-0.26	0.07	0.90*
Physical Activity Participation	-0.38	-0.02	0.09	-0.19	0.88*
Self-Rated Health	0.10	-0.24	0.01	0.93*	0.10
Scuba Diving Participation	-0.25	-0.87*	-0.26	0.05	0.31
Stress and Anxiety	-0.65*	0.71*	-0.16	0.01	0.01
Body Mass Index	0.84*	0.14	-0.44	0.15	-0.06
Handgrip Strength	0.89*	-0.11	0.11	0.30	-0.28
Mean Arterial Pressure	-0.19	0.89*	0.24	-0.09	-0.01
Mean Heart Rate	-0.07	0.22	0.94*	-0.02	-0.10
Total Ectopic	-0.23	0.60*	0.08	-0.34	0.64*
Respiratory Sinus Arrhythmia	0.00	0.00	0.00	0.00	0.00
Variance Explained	24.4%	20.5%	20.0%	17.7%	17.5%

*Significant factor loading (|>0.50|).

Note: PC=principal component.

Supplementary Table S3. Factor Loadings for the Principal Component Analysis in Persons without Weakness.

Variable	PC1	PC2	PC3	PC4	PC5	PC6	PC7
Age	-0.36	-0.77*	0.06	-0.09	0.04	-0.25	0.06
Gender	-0.90*	0.09	-0.04	0.05	-0.04	-0.02	0.06
Spicy Food Intake	0.22	0.06	0.01	-0.15	0.69*	-0.07	0.18
Sodium Intake	0.20	-0.01	-0.26	-0.01	0.17	0.76*	0.12
Caffeine Intake	0.13	-0.08	0.55*	0.23	0.15	-0.06	0.25
Processed Meat Intake	0.34	0.16	-0.50*	0.36	0.21	0.07	-0.08
Nicotine Intake	0.02	-0.09	-0.05	0.78*	-0.13	0.11	0.01
Sleep	-0.14	-0.18	0.02	0.10	0.72*	0.13	-0.22
Physical Activity Participation	-0.23	-0.01	0.31	0.72*	0.10	-0.18	0.11
Self-Rated Health	0.09	-0.03	0.73*	0.03	-0.03	0.06	-0.15
Scuba Diving Participation	0.37	-0.02	0.04	0.01	-0.17	-0.01	-0.62*
Stress and Anxiety	-0.12	0.67*	0.31	-0.08	0.12	-0.07	0.16
Body Mass Index	0.06	-0.53*	0.32	0.09	0.16	-0.01	0.04
Handgrip Strength	0.90*	0.13	0.06	-0.06	0.04	0.01	-0.01
Mean Arterial Pressure	0.27	-0.47	-0.07	-0.31	-0.13	-0.20	0.46
Mean Heart Rate	0.10	-0.21	0.10	0.29	-0.26	0.14	0.59*

Total Ectopic	0.16	-0.16	-0.34	-0.04	0.12	-0.69*	0.06
Respiratory Sinus Arrhythmia	0.05	0.76*	-0.15	-0.07	-0.05	0.01	-0.19
Variance Explained	20.0%	19.7%	14.0%	13.6%	11.3%	10.9%	10.6%

*Significant factor loading (|>0.50|). *Note*: PC=principal component.

Supplementary File S1. Self-Report Questionnaire.†
†Items were pre-specified for inclusion in this investigation.

This questionnaire will ask you questions relating to possible risk factors for arrythmias (irregular heart rhythms). If you have questions, ask us for help.

The following section asks questions about your demographics:

1. Please circle the race that you most identify with:
    a. American Indian/Alaska Native
    b. Asian
    c. Native Hawaiian or Other Pacific Islander
    d. Black or African American
    e. White
    f. More than One Race
    g. Other, please detail:
2. What is your age:
3. Please circle your marital status:
    a. Single
    b. Married
    c. Widowed
    d. Other, please detail:
4. What gender do you identify with?
    a. Male
    b. Female
5. Please circle the highest level of education you have completed:
    a. Some High School
    b. High School Graduate/GED or Equivalent
    c. Some college or Vocational Training
    d. Completed Associate Degree
    e. Completed Bachelor Degree
    f. Completed Graduate Degree

The following section asks questions about your current diet/nutrition:

1. Do you consume food that is considered hot/spicy at least once a week?
    a. Yes
    b. No
2. On a daily basis, do you consume > 100% daily value of sodium (>2300 mg/day)?
    a. Yes
    b. No
3. Do you regularly consume more than 400 mg of caffeine a day (about 4 cups of brewed coffee, also see accompanying list of caffeinated items)?
    a. Yes
    b. No
4. Do you consume "red meats" (beef, pork, etc.) more than once a week?
    a. Yes

    b. No
5. Do you consume processed meats (Deli meats, hot dogs, bologna, etc.) more than once a week?
    a. Yes
    b. No

The following section asks questions about your current lifestyle and activity levels:

1. Do you currently smoke cigarettes?
    a. Yes
    b. No
2. Do you currently use any nicotine replacement products (gum, patches, lozenges, etc.)?
    a. Yes
    b. No
3. Do you currently vape or use e-cigarettes?
    a. Yes
    b. No
4. Do you get 7 or more hours of sleep per night in a week?
    a. Yes
    b. No
5. Do you regularly get 150 minutes or more of moderate physical activity exercise per week?
    a. Yes
    b. No
6. Prior to the past 12 months (and whether currently or not) did you previously in your life engage in aquatic activities such as swimming, scuba diving, etc.?
    a. Yes
    b. No
7. How would you rate your overall health?
    a. Excellent
    b. Very good
    c. Good
    d. Fair
    e. Poor

The following section asks questions about your current well-being/emotional state:

1. Would you consider yourself a high-stress individual?
    a. Yes
    b. No
2. Would you describe yourself as an anxious person?
    a. Yes
    b. No
